# Comparative differences in the risk of major gastrointestinal bleeding among different direct oral anticoagulants: An updated traditional and Bayesian network meta-analysis

**DOI:** 10.3389/fphar.2022.1049283

**Published:** 2023-01-04

**Authors:** Xiuehui Chen, Lili Wang, Huijun Li, Weichao Huang, Lingyue Zhao, Wenqin Guo

**Affiliations:** ^1^ Longhua District Central Hospital, Shenzhen, China; ^2^ Fuwai Hospital Chinese Academy of Medical Sciences, Shenzhen, China; ^3^ Huazhong University of Science and Technology Union Shenzhen Hospital, Shenzhen, China

**Keywords:** randomized controlled trial, major gastrointestinal bleeding, novel oral anticoagulants, meta-analysis, network meta-analysis

## Abstract

**Background:** The most favorable gastrointestinal (GI) bleeding safety profile among different types of direct oral anticoagulants (DOACs) remains controversial. This meta-analysis includes the latest studies and aims to compare GI bleeding risk associated with the use of various DOACs.

**Methods:** PubMed, Cochrane library, and clinicaltrial.gov were searched. Randomized control trials (RCTs) evaluating the safety of DOACs were identified. The primary endpoint assessed was major GI bleeding.

**Results:** A total of 37 RCTs were included in the analyses. Based on the traditional meta-analysis, the major GI bleeding risk was different among various DOACs (interactive *p*-value <.10). Network meta-analysis findings showed that no DOACs increased the risk of major GI bleeding compared with conventional therapy. Furthermore, a 10 mg daily administration of apixaban reduced the major GI bleeding risk more than daily doses of 60 mg edoxaban, ≥15 mg rivaroxaban, and 300 mg dabigatran etexilate. No difference was observed between daily doses of 300 mg dabigatran etexilate, 60 mg edoxaban, and ≥15 mg rivaroxaban. The major GI bleeding risk associated with 30 mg daily dose of edoxaban was lower than with 10 mg daily rivaroxaban, and no differences between daily 5 mg apixaban, 30 mg edoxaban, and 220 mg dabigatran etexilate were observed.

**Conclusion:** Differences in the major GI bleeding risk were observed when various DOACs were compared. Among standard-dose DOACs, apixaban was associated with the lowest degree of major GI risk. Among low-dose DOACs, edoxaban was associated with a lower major GI bleeding risk than rivaroxaban.

## Introduction

Indications for the use of direct oral anticoagulants (DOACs) are widening to include stroke prevention in those with high-risk atrial fibrillation (AF) and venous thromboembolism prevention and treatment (Kearon, et al., 2016; Steffel, et al., 2021). Although DOACs reduce thromboembolism risk, the risk of concomitant bleeding—especially major gastrointestinal (GI) bleeding—is of clinical concern. The occurrence of major GI bleeding reduces medication compliance and increases thromboembolism and even mortality risk (Deitelzweig, et al., 2021).

Previous studies have evaluated the relationship between DOAC use and major GI bleeding. A prior meta-analysis showed that major GI bleeding risk was not elevated in those receiving DOACs compared with a control group (Miller, et al., 2017). Furthermore, another meta-analysis revealed that, unlike other types of DOACs, rivaroxaban significantly increased major GI bleeding risk (Gu, et al., 2020). Rivaroxaban use was associated with increased rates of GI bleeding compared with apixaban and dabigatran etexilate use, regardless of the indication (Ingason, et al., 2021). A network meta-analysis assessed major GI bleeding risk differences associated with different dosages and types of NOAC therapy; its results showed that major GI bleeding risk associated with 5 mg BID apixaban was lower than that with 20 mg QD rivaroxaban and 150 mg BID dabigatran etexilate (Radadiya, et al., 2021). We conducted a network meta-analysis that showed that both apixaban and edoxaban were associated with a significantly reduced risk of major GI bleeding compared with dabigatran etexilate and rivaroxaban, suggesting that apixaban and edoxaban have optimal major GI bleeding safety profiles, while the use of rivaroxaban and dabigatran etexilate is less safe (Guo, et al., 2019).

Controversy regarding major GI bleeding risk differences among different dosages and types of DOACs remains. Studies published until 2019 were considered in previous meta-analyses (Guo, et al., 2019; Radadiya, et al., 2021). In the past three years, additional work has evaluated the efficacy and safety of DOACs in the treatment of disease. Therefore, we aimed to perform an updated network meta-analysis that includes the latest studies to comprehensively evaluate GI bleeding risk differences among patients receiving various DOACs.

## Materials and methods

We report this meta-analysis in accordance with Preferred Reporting Items for Systematic Reviews and Meta-Analyses guidelines (Moher, et al., 2009).

### Research strategy

We searched PubMed, Cochran Library, clinicaltrial.gov and reference lists of relevant papers. The following keywords were used for searches: “apixaban,” “edoxaban,” “rivaroxaban,” and “dabigatran etexilate.” Studies published up to 1 April 2022, were considered.

### Inclusion and exclusion criteria

The inclusion criteria were: 1) the intervention was a DOAC (apixaban, rivaroxaban, dabigatran etexilate, and edoxaban); 2) control treatment of a DOAC, placebo, conventional treatment regimens including vitamin K antagonists (VKA), low molecular weight heparin, or aspirin; 3) major GI bleeding reported as an outcome; 4) randomized controlled trials. If there were multiple reports from the same study, the most recently published that reported major GI bleeding events was included. The exclusion criteria were: 1) studies that included cancer patients, because patients with GI tumors were at increased risk of GI bleeding; 2) studies that included very elderly patients with mean ages greater than 85 years; 3) the background therapy included an antiplatelet regimen; 4) the intervention was not a DOAC or the intervention was a DOAC combined with antiplatelet therapy; 5) studies not reporting major GI bleeding events; 6) observational studies or research letters.

### Data extraction and assessment of primary outcome and study quality

Two researchers designed a table that was used for data extraction. The table was then filled with relevant information using data from the included studies. In the case of a dispute between the two researchers regarding the process of information extraction, a third researcher was consulted. The primary endpoint of the study was major GI bleeding, which was defined according to the individual studies. The Cochrane risk-of-bias tool for randomized trials was used to evaluate the quality of all included studies (Higgins, et al., 2011).

### Statistical analysis

#### Meta-analysis

When performing a traditional meta-analysis, odds ratio (OR) and 95% confidence intervals (CIs) were used to measure the effect size for dichotomous data. The significance threshold was set at *p*-value <.05. Considering the potential for heterogeneity among the studies, we used the DerSimonian-Laird random-effects model to assess data when performing a traditional meta-analysis (DerSimonian and Laird, 1986). Heterogeneity between studies was analyzed using I^2^-statistic and Cochran Q tests [10]. I^2^ values <25%, ≥25% and <50%, ≥50% and <75%, and ≥75% indicated no, low, moderate, and high heterogeneity among studies, respectively (Higgins, et al., 2003). The subgroup analysis was performed based on the dose and type of the DOACs prescribed. An interaction test was performed to evaluate differences among DOACs. Values of *p* < .1 suggested that differences among subgroups were statistically significant. An R software meta package (version 4.0.3) was used.

#### Network meta-analysis

In the network meta-analysis, ORs and 95% credible intervals (CrIs) were used to assess the effect size. A CrI that did not include one indicated statistically significant differences. A Bayesian random-effects model was used for the analysis and a Markov Chain–Monte Carlo method was used for Bayesian inference (Dias, et al., 2013a). We used informative variance prior to generating the posterior distributions of the model parameters and fitted three chains, yielding 300,000 iterations (100,000 per chain) (Turner, et al., 2012; Wolfe, et al., 2018). Convergence was assessed by the Brooks–Gelman–Rubin diagnostic (Brooks and Gelman, 1998). Model fit was assessed with a deviance information criterion (Dias, et al., 2013b). Node-splitting was used to evaluate inconsistency between direct and indirect evidence and for *p*-value calculation (Dias, et al., 2010). Values of *p* < .05 indicated inconsistencies between direct and indirect evidence. To evaluate the association between population and study characteristics and the clinical outcome, we performed a network meta-regression analysis using mean age, proportion of males, body mass index (BMI), body weight, proportion of Asians, indication, and follow-up time as variables (Dias, et al., 2013a). Publication bias was assessed by drawing a comparison-adjusted funnel plot (Salanti, et al., 2011). The network meta-analysis was performed using gemtc and rjags R software (version 4.0.3) and STATA13.1 packages.

## Results

**FIGURE 1:**
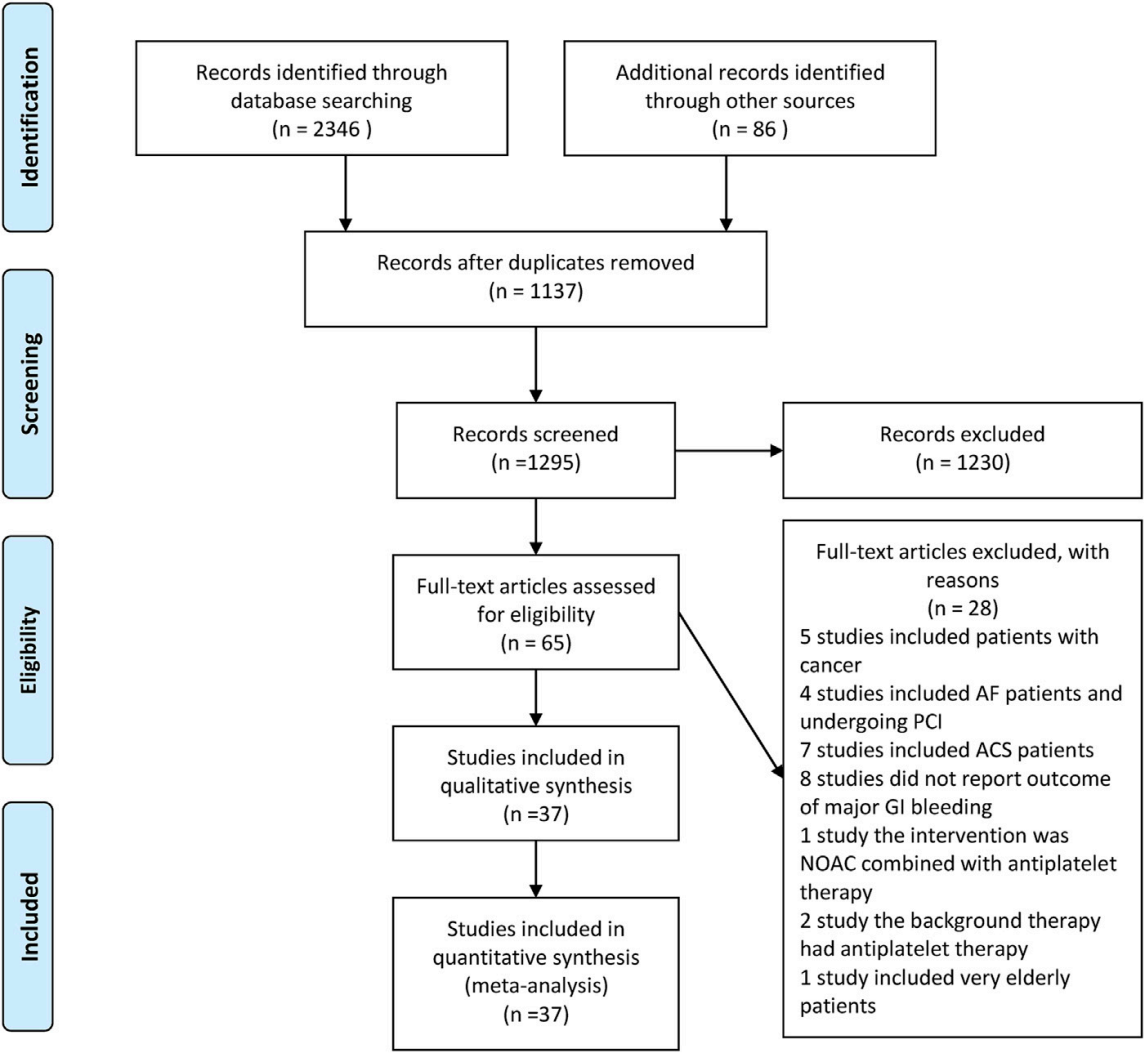
Flowchart of study screening.

A flow chart depicting the literature search strategy is shown in Figure 1. In total, 2,346 studies were identified through database searching and 86 were identified through other sources. After reading the title and abstract, 1,230 studies were eliminated, and the remaining 65 were downloaded; the full text of each article was read. After reading the full text of these, 28 studies were excluded. The reasons for excluding the studies are shown in the Supplementary Appendix.

**FIGURE 2:**
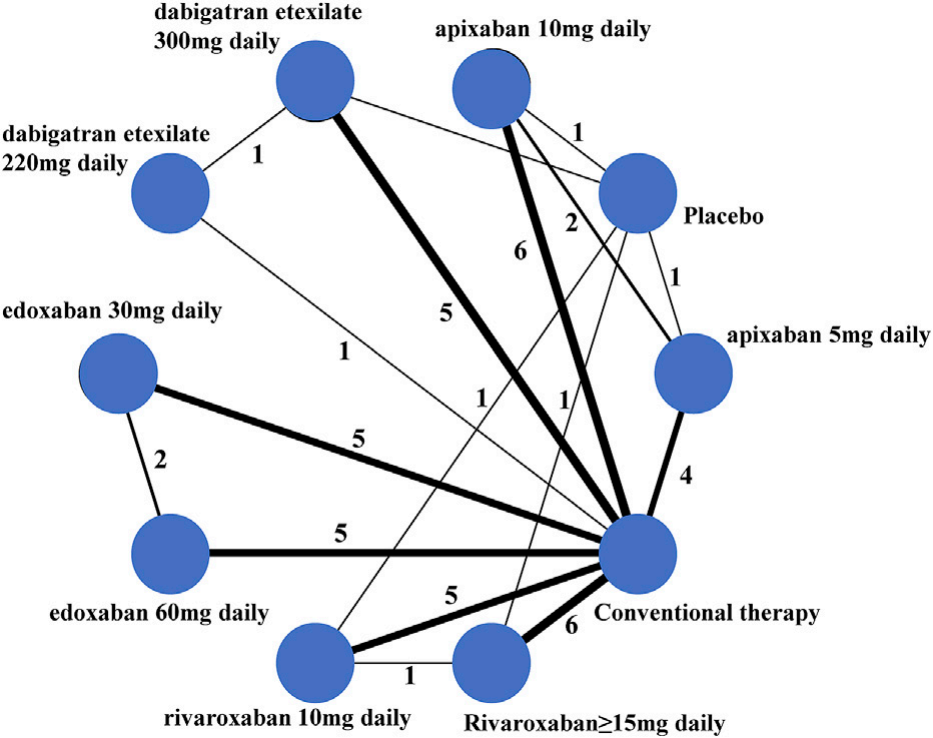
Network plot for the Bayesian network meta-analysis. Each node represents a regimen, the lines between the nodes represent the head-to-head comparisons, the thickness of the lines represents the number of studies, and the number of studies is marked beside.

In the end, 37 studies were included in the meta-analysis (references to the included studies are shown in Supplementary Appendix). Among these, six were three-arm trials (Eriksson, et al., 2006; Connolly, et al., 2009; Chung, et al., 2011; Ogawa, et al., 2011; Agnelli, et al., 2013; Giugliano, et al., 2013). From the three-arm COMPASS study, the 5 mg BID rivaroxaban and aspirin-alone groups were included in the analysis, based on the inclusion and exclusion criteria (Eikelboom, et al., 2017). A network plot is shown in Figure 2. The characteristics of the included studies and each study population are shown in Table 1. The international normalized ratio (INR) values of 2*–*3 were maintained among patients taking VKA in most studies; however, in Japanese studies, INR values ranging from 1.5 to 2.5 were observed. The mean age, proportion of males and Asians, BMI, weight, and follow-up time varied widely among the studies. Definitions of “major bleeding” used in each individual study are shown inSupplementary Table S1. A Cochrane risk-of-bias assessment is shown in Supplementary Figure S1. The results showed that most studies were of high quality.

**TABLE 1:** Characteristics of the included studies and populations.

Study	Year	Intervention	Dose	Control	Dose	Sample size	INR	Follow-up time	Mean age (y)	Male (%)	BMI (kg/m^2^)	Weight (kg)	DM (%)	Asian (%)	Indication for taking NOCA
ADVANCE	2009	Apixaban	5 mg daily	Enoxaparin	60 mg daily	3,184	NA	12 days	65.8	37.9	31	86.7	NA	0.9	Thromboprophylaxis
ADVANCE-2	2010	Apixaban	5 mg daily	Enoxaparin	40 mg daily	3,009	NA	12 days	NA	27.5	29.1	78	NA	15	Thromboprophylaxis
ADVANCE-3	2010	Apixaban	5 mg daily	Enoxaparin	40 mg daily	5,332	NA	35 days	60.8	46.7	28	79.7	NA	5.7	Thromboprophylaxis
AMPLIFY EXT	2013	Apixaban	5 mg daily	Placebo	NA	1,669	NA	12 months	56.7	57.4	NA	85.4	NA	4.4	VTE
		Apixaban	10 mg daily												
AMPLIFY	2013	Apixaban	10 mg daily	Warfarin	NA	5,395	2-3	6 months	57	58.7	NA	84.6	NA	8.4	VTE
ARISTOTLE	2011	Apixaban	10 mg daily	Warfarin	NA	18,140	2-3	1.8 years	NA	64.7	NA	82	25	14.5	AF
AVERROES	2011	Apixaban	10 mg daily	Aspirin	81–324 mg daily	5,599	NA	1.1 years	70	58.5	28	NA	20	19.4	AF
EMANATE	2018	Apixaban	10 mg daily	Warfarin	NA	1,456	2-3	90 days	64.6	66.8	NA	NA	19.6	10.3	AF and cardioversion
AMPLIFY-J	2015	Apixaban	10 mg daily	Warfarin	NA	80	1.5–2.5	23 weeks	65.2	48.8	23.7	61.4	11.3	100	VTE
ARISTOTLE-J	2011	Apixaban	5 mg daily	Warfarin	NA	222	2-3	12 weeks	70.3	62	24.7	65.8	23.4	100	AF
		Apixaban	10 mg daily												
RE-COVER	2009	Dabigatran etexilate	300 mg daily	Warfarin	NA	2,539	2-3	163 days	NA	58.4	28.6	84.9	NA	2.6	VTE
RE-COVER II	2014	Dabigatran etexilate	300 mg daily	Warfarin	NA	2,568	2-3	164 days	NA	60.6	28.4	83	NA	21	VTE
RE-MEDY	2013	Dabigatran etexilate	300 mg daily	Warfarin	NA	2,856	2-3	473 days	54.7	60.9	NA	86	9	8	VTE
RE-SONATE	2013	Dabigatran etexilate	300 mg daily	Placebo	NA	1,343	NA	164 days	55.8	55.5	NA	83.8	8	8.9	VTE
RELY	2009	Dabigatran etexilate	220 mg daily	Warfarin	NA	12,098	2-3	2 years	71.5	63.6	NA	82.7	23.3	15.9	AF
		Dabigatran etexilate	300 mg daily												
RE-CIRCUIT	2017	Dabigatran etexilate	300 mg daily	Warfarin	NA	635	2-3	9 weeks	59.2	74.8	28.6	NA	10.1	NA	AF and ablation
RE-SPECT ESUS	2019	Dabigatran etexilate	300 mg daily	Aspirin	100 mg daily	5,390	NA	19 months	64.2	63.2	27.2	NA	22.7	22.8	ESUS
Chung, et al	2011	Edoxaban	30 mg daily	Warfarin	NA	154	2-3	3 months	65.1	65.4	NA	70	29.5	100	AF
		Edoxaban	60 mg daily												
ENGAGE-AF-TIMI 48	2013	Edoxaban	30 mg daily	Warfarin	NA	21,026	2-3	2.8years	NA	61.9	NA	NA	36.1	13.8	AF
		Edoxaban	60 mg daily												
Fuji, et al	2014	Edoxaban	30 mg daily	Enoxaparin	40 mg daily	88	NA	12 days	76.1	20.5	NA	53.7	NA	100	Thromboprophylaxis
Hakusai-VTE	2013	Edoxaban	60 mg daily	Warfarin	NA	8,240	2-3	250 days	55.8	57.2	NA	NA	NA	21	VTE
STARS E-3	2014	Edoxaban	30 mg daily	Enoxaparin	40 mg daily	703	NA	13 days	72.3	20.2	NA	60.2	NA	100	Thromboprophylaxis
STARS J-V	2015	Edoxaban	30 mg daily	Enoxaparin	40 mg daily	604	NA	13 days	62.8	14.1	24.3	57.3	NA	100	Thromboprophylaxis
ENVISAGE-TAVI AF	2021	Edoxaban	60 mg daily	Warfarin	NA	1,426	2-3	554 days	82.1	52.5	27.7	75.3	36.9	12.7	TAVI
ENSURE-AF	2016	Edoxaban	60 mg daily	Warfarin	NA	2,149	2-3	58 days	64.2	65	30.6	91	19	0	AF and cardioversion
COMPASS	2017	Rivaroxaban	10 mg daily	Aspirin	100 mg daily	18,243	NA	23 months	68.2	22	28.3	NA	37.8	15.4	CAD/PAD
EINSTEIN	2010	Rivaroxaban	20 mg daily	Placebo	NA	1,188	NA	264 days	58.3	56.8	NA	NA	NA	NA	VTE
J-ROCKET AF	2012	Rivaroxaban	15 mg daily	Warfarin	NA	1,278	2-3	30 months	71.1	80.6	NA	NA	38	100	AF
RECORD1	2008	Rivaroxaban	10 mg daily	Enoxaparin	40 mg daily	4,433	NA	35 days	63.2	44.5	27.8	78.2	NA	0.1	Thromboprophylaxis
RECORD2	2008	Rivaroxaban	10 mg daily	Enoxaparin	40 mg daily	2,457	NA	35 days	61.5	46.4	27	74.7	NA	20	Thromboprophylaxis
RECORD4	2009	Rivaroxaban	10 mg daily	Enoxaparin	60 mg daily	3,034	NA	12 days	NA	34.9	30.8	84.5	NA	19	Thromboprophylaxis
ROCKET AF	2011	Rivaroxaban	20 mg daily	Warfarin	NA	14,236	2-3	590 days	NA	60.3	28.2	NA	40	7.4	AF
NAVIGATE ESUS	2018	Rivaroxaban	15 mg daily	Aspirin	100 mg daily	7,213	NA	11 months	66.9	61	27.2	NA	25	19	ESUS
ODIXa-HIP	2006	Rivaroxaban	10 mg daily	Enoxaparin	40 mg daily	401	NA	60 days	NA	43.1	27	78	NA	0	Thromboprophylaxis
		Rivaroxaban	20 mg daily												
X-VeRT	2014	Rivaroxaban	20 mg daily	Warfarin	NA	1,487	2-3	30 days	64.8	72.8	30.14	NA	20.4	5.3	AF and cardioversion
J-EINSTEIN	2015	Rivaroxaban	15 mg daily	Warfarin	NA	59	1.5–2.5	12 months	65.7	52.6	NA	NA	NA	100	VTE
ERIKA	2016	Rivaroxaban	10 mg daily	Placebo	NA	234	NA	3 months	45.4	67.2	27.9	NA	1.6	100	Thromboprophylaxis

VTE, venous thromboembolism; AF, atrial fibrillation; CAD, coronary artery disease; PAD, peripheral artery disease; CT, conventional therapy; TAVI, transcatheter aortic valve implantation; ESUS, embolic stroke of undetermined source.

### Meta-analysis

**FIGURE 3:**
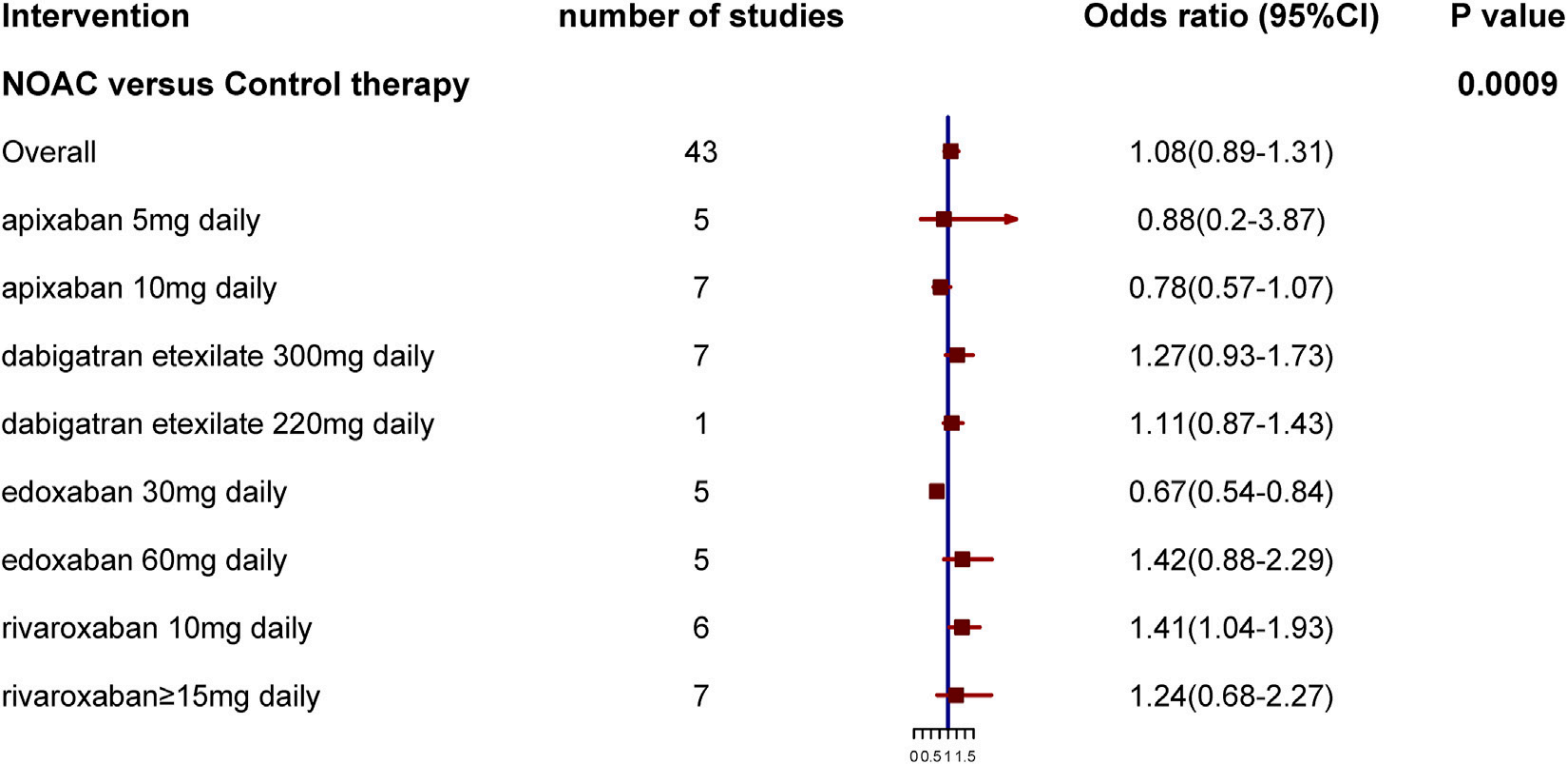
Results of subgroup analysis based on dosages and type of direct oral anticoagulants.

Compared with the control groups, DOACs did not increase the risk of major GI bleeding (OR: 1.08, 95% CI: .89–1.31, *p* = .4206, I^2^ = 51%) (shown in Figure 3; Supplemental Figure S2). However, subgroup analysis based on dosages and types of DOACs revealed that, compared with controls, 30 mg daily edoxaban significantly reduced (OR: .67, 95% CI: 0.54–.84, I^2^ = 0%), whereas 10 mg daily rivaroxaban significantly increased, major GI bleeding risk (OR: 1.41, 95% CI: 1.04–1.93, I^2^ = 0%). No differences in GI bleeding risk were observed when other DOACs and controls were compared. Significant differences in major GI bleeding risk were observed among patients receiving different DOACs (interactive *p*-value = .0009).

### Network meta-analysis

The results of this network meta-analysis are shown in Table 2. No DOACs were associated with an increased risk of major GI bleeding compared with conventional therapy. The doses of 300 mg daily dabigatran etexilate, 60 mg daily edoxaban, 10 mg daily rivaroxaban, and ≥15 mg daily rivaroxaban were associated with a higher major GI bleeding risk than the placebo. Among standard-dose DOACs, the risk of major GI bleeding associated with 10 mg daily apixaban was significantly reduced compared with 300 mg daily dabigatran etexilate, 60 mg daily edoxaban, and ≥15 mg daily rivaroxaban (OR: .58, 95% CrI: .34–.97 for dabigatran etexilate, OR: .53, 95% CrI: .28–.86 for edoxaban, and OR: .55, 95% CrI: .31–.93 for rivaroxaban, respectively). The degree of risk associated with 300 mg daily dabigatran etexilate, 60 mg daily edoxaban, and ≥15 mg daily rivaroxaban did not differ.

**TABLE 2:** Estimated relative treatment effects as odds ratio (OR) and corresponding 95% credible intervals.

	Apixaban 5 mg daily	Placebo	Apixaban 10 mg daily	Dabigatran etexilate 300 mg daily	Dabigatran etexilate 220 mg daily	Edoxaban 30 mg daily	Edoxaban 60 mg daily	Rivaroxaban 10 mg daily	Rivaroxaban ≥15 mg daily	Conventional therapy
Apixaban 5 mg daily	-	.32 (.04, 1.73)	.88 (.31, 2.42)	1.52 (.54, 4.29)	1.22 (.41, 3.62)	.87 (.30, 2.6)	1.69 (.6, 4.90)	1.78 (.60, 5.53)	1.60 (.57, 4.57)	1.21 (.46, 3.22)
Placebo	3.1 (.58, 25.6)	-	2.7 (.58, 2.76)	**4.69 (1.02, 36.16)**	3.76 (.78, 3.03)	2.69 (.55, 21.66)	**5.25 (1.1, 41.3)**	**5.56 (1.12, 45.43)**	**4.96 (1.08, 37.86)**	3.73 (.83, 28.39)
Apixaban 10 mg daily	1.14 (.41, 3.19)	.37 (.05, 1.72)	-	**1.73 (1.03, 2.95)**	1.38 (.75, 2.74)	.97 (.55, 1.99)	**1.90 (1.16, 3.59)**	**2.00 (1.09, 4.42)**	**1.82 (1.08, 3.26)**	1.36 (.95, 2.17)
Dabigatran etexilate 300 mg daily	.66 (.23, 1.85)	**.21 (.03, .98)**	**.58 (.34, .97)**	-	.79 (.5, 1.37)	.56 (.33, 1.11)	1.1 (.69, 2.02)	1.16 (.65, 2.44)	1.05 (.64, 1.80)	.79 (.58, 1.20)
Dabigatran etexilate 220 mg daily	.82 (.28, 2.46)	.27 (.03, 1.29)	.73 (.37, 1.34)	1.26 (.73, 2.02)	-	.71 (.36, 1.5)	1.38 (.75, 2.75)	1.45 (.73, 3.29)	1.33 (.68, 2.48)	.99 (.61, 1.71)
Edoxaban 30 mg daily	1.15 (.38, 3.38)	.37 (.05, 1.83)	1.03 (.50, 1.82)	1.78 (.9, 3.06)	1.41 (.67, 2.79)	-	**1.94 (1.18, 3.24)**	**2.04 (1.01, 4.39)**	1.87 (.95, 3.36)	1.39 (.85, 2.29)
Edoxaban 60 mg daily	.59 (.20, 1.67)	**.19 (.02, .91)**	**.53 (.28, .86)**	.91 (.5, 1.46)	.73 (.36, 1.34)	**.51 (.31, .85)**	-	1.05 (.55, 2.11)	.96 (.52, 1.6)	.72 (.48, 1.06)
Rivaroxaban 10 mg daily	.56 (.18, 1.66)	**.18 (.02, .89)**	**.5 (.23, .92)**	.86 (.41, 1.54)	.69 (.3, 1.37)	**.49 (.23, .99)**	.95 (.47, 1.83)	-	.91 (.43, 1.68)	.68 (.38, 1.14)
Rivaroxaban≥15 mg daily	.62 (.22, 1.76)	**.20 (.03, .93)**	**.55 (.31, .93)**	.95 (.56, 1.57)	.75 (.4, 1.46)	.53 (.30, 1.05)	1.04 (.63, 1.92)	1.09 (.60, 2.33)	-	.74 (.52, 1.15)
Conventional therapy	.83 (.31, 2.17)	.27 (.04, 1.21)	.73 (.46, 1.05)	1.27 (.84, 1.74)	1.01 (.59, 1.64)	.72 (.44, 1.18)	1.40 (.94, 2.10)	1.47 (.88, 2.62)	1.34 (.87, 1.91)	-

The bold value means that the outcome has statistical significance.

Among low-dose DOACs, the risk of major GI bleeding associated with 30 mg daily edoxaban was lower than that with 10 mg daily rivaroxaban (OR: .49, 95% CrI: .23–.99). There were no differences between individuals administered with 5 mg daily apixaban, 10 mg daily rivaroxaban, and 220 mg daily dabigatran etexilate, nor between those who received 30 mg daily edoxaban, 5 mg daily apixaban, and 220 mg daily dabigatran etexilate.

Additionally, the dose of 10 mg daily apixaban had a lower major GI bleeding risk than 10 mg daily rivaroxaban. The major GI bleeding risk of 30 mg daily edoxaban was lower than that of 60 mg daily edoxaban.

No inconsistency was observed between direct and indirect evidence (Supplementary Table S2). The results of our network meta-regression analysis showed that clinical outcomes were independent of mean age, BMI, body weight, proportion of males, proportion of Asians, indication, and follow-up time (Supplementary Table S3). A comparison-adjusted funnel plot suggested no publication bias (Supplementary Figure S3).

## Discussion

The main findings of the traditional meta-analysis showed that rates of major GI bleeding differ among groups administered with different doses and types of DOACs (*p* < .1). The network meta-analysis showed that 10 mg daily apixaban was associated with a lower degree of major GI bleeding risk than 300 mg daily dabigatran etexilate, ≥15 mg daily rivaroxaban, and 60 mg daily edoxaban. No between-group differences in bleeding risk associated with 300 mg daily dabigatran etexilate, ≥15 mg daily rivaroxaban, and 60 mg daily edoxaban were observed. The risk of major GI bleeding was reduced in those administered with 30 mg daily edoxaban compared with those administered with 10 mg daily rivaroxaban; no major GI bleeding risk differences between patients administered with 5 mg daily apixaban, 30 mg daily edoxaban, and 220 mg daily dabigatran etexilate were observed.

Differences in the major GI bleeding risk among different DOACs have been observed in previous studies. The COMPASS study evaluated the efficacy and safety of low-dose NOAC use for preventing stable atherosclerotic cardiovascular disease. The risk of major GI bleeding with 10 mg QD rivaroxaban alone was significantly greater than that with aspirin alone (Eikelboom, et al., 2017). The RE-Ly study, which evaluated the use of dabigatran etexilate for the prevention of stroke in AF patients showed that 150 mg and 110 mg BID dabigatran etexilate increased the risk of major GI bleeding compared with warfarin. Furthermore, major GI bleeding risk was significantly higher with dabigatran etexilate 150 mg BID than with 110 mg BID (Connolly, et al., 2009). The ENGAGE AF-TIMI study evaluated the efficacy and safety of edoxaban use for preventing stroke in AF patients (Giugliano, et al., 2013). Its results showed that 60 mg QD edoxaban was associated with a greater degree of major GI bleeding risk than warfarin, while 30 mg QD edoxaban showed lower major GI bleeding risk compared with warfarin. Our study integrated all current research on DOAC use and indirectly compared differences in the major GI bleeding risk associated with different dosages and types of DOACs. The results showed that, among patients administered standard-dose DOACs, those who received apixaban had the lowest risk of major GI bleeding. Furthermore, among those administered with low-dose DOACs, major GI bleeding risk associated with edoxaban was lower than with rivaroxaban. Indeed, the DOACs were commonly used to reduce ischemic events. Nevertheless, it also increased bleeding risk, which causes confusion for clinicians. Our study suggested that major GI bleeding risk differed across DOACs. These findings are important because the drug with the most favorable GI bleeding safety profile should be preferred, especially for treating those at high risks of GI bleeding.

The different bleeding risks among DOACs may be related to the pharmacokinetics of the drugs. For example, rivaroxaban and edoxaban are administered once daily, while dabigatran etexilate and apixaban are administered twice daily, with higher peak plasma concentrations for the former than for the latter. Two previous studies have shown that the peak plasma concentration of rivaroxaban is almost twice that of apixaban, and that the anti-Xa activity is highly correlated with plasma concentrations, such as rivaroxaban with higher maximum anti-Xa activity and higher 24-h area under the curve for anti-Xa activity (Frost, et al., 2014; Kreutz, et al., 2017). However, the above mechanisms still do not fully explain the different bleeding risks between apixaban and dabigatran etexilate as both were administered twice daily. Further studies are needed to elucidate the mechanisms underlying the differences in major GI bleeding risks among DOACs.

### Comparison with other studies

In agreement with evidence from previously reported studies, major GI bleeding risk differences were observed among DOACs. In patients with AF, apixaban better reduced the risk of major GI bleeding than rivaroxaban and dabigatran etexilate, and rivaroxaban was associated with a higher risk of major GI bleeding compared with dabigatran etexilate (Abraham, et al., 2017). Rivaroxaban increases the risk of GI bleeding compared with dabigatran etexilate and apixaban, a finding that was independent of the indication for use (Ingason, et al., 2021). However, the above studies were retrospective with the potential for selection bias. Furthermore, their data were limited to those contained within databases, which were limited regarding edoxaban bleeding risk assessment. To date, no studies have directly compared GI bleeding risks amongst individual DOACs due to the very large sample sizes required to perform such studies. Network meta-analyses may be used to indirectly compare the safety of different regimens; thus, performing a network meta-analysis that compares the bleeding risks of different types of DOACs is of great importance. We previously performed a network meta-analysis to evaluate the GI bleeding risk differences among different DOACs. Our findings revealed that apixaban and edoxaban have favorable GI bleeding safety profiles, unlike rivaroxaban and dabigatran etexilate (Guo, et al., 2019). Another study showed that standard-dose apixaban reduced the risk of major GI bleeding compared with standard-dose rivaroxaban and dabigatran etexilate; however, no favorable GI bleeding safety profile was observed with regard to edoxaban use (Radadiya, et al., 2021). Notably, only studies published before 2019 were considered in the above studies. Since then, more studies evaluating the efficacy and safety of DOACs in the treatment of disease have been reported. After including such studies, we found that standard-dose apixaban was associated with a lower risk of major GI bleeding than standard-dose rivaroxaban and dabigatran etexilate; this was similar to the results of the previous meta-analysis. However, our study also found that standard-dose apixaban was associated with a lower major GI risk than standard-dose edoxaban. Additionally, we also compared the major GI bleeding risks between low-dose DOACs, which showed that low-dose edoxaban is associated with a lower degree of GI bleeding risk compared with low-dose rivaroxaban. These findings are additional to the results of the previous meta-analysis.

### Study limitations

Our study has some limitations. First, we included studies with different indications for treatment, which may have resulted in bleeding risk differences among the populations considered, thereby increasing study heterogeneity. However, our study excluded studies that recruited patients with cancer, the very elderly, and those receiving antiplatelet background therapy, to minimize the heterogeneity between studies. Furthermore, a random effect model was used to combine evidence. Second, a wide ranging mean age, a prevalence of males and Asians, and follow-up time data also increased heterogeneity between studies. However, we used network meta-regression analysis to evaluate the association between study/population characteristics and clinical outcomes. The analysis revealed that the clinical outcome was independent of study/population characteristics. Third, recent studies have suggested that patients with prior GI bleeding could benefit from DOACs rather than warfarin (Lip, et al., 2021). Our study was based on the study level, and limited data were available regarding patients with prior GI bleeding; therefore, we could not compare GI bleeding risks associated with different DOACs in patients with prior GI bleeding.

## Conclusion

This study revealed that DOAC dosage and type affect major GI bleeding risk. Among standard-dose DOACs, apixaban use was associated with the lowest degree of major GI bleeding risk. Among low-dose DOACs, edoxaban was associated with lower major GI bleeding risk than rivaroxaban.

## Abbreviations

AF, atrial fibrillation; BMI, body mass index; CIs, confidence intervals; CrI, credible interval; DOACs, direct oral anticoagulants; GI, gastrointestinal; INR, international normalized ratio; OR, odds ratio; VKA, vitamin K antagonists.
