# Supplementary material for: Comparative differences in the risk of major gastrointestinal bleeding among different direct oral anticoagulants: An updated traditional and Bayesian network meta-analysis
Source: Front Pharmacol. 2023 Jan 4;13:1049283. doi: 10.3389/fphar.2022.1049283 (PMC9845702; doi:10.3389/fphar.2022.1049283)
Supplement: Supplementary file 1 [file DataSheet1.PDF]

Supplemental File

Supplemental Appendix. The references of including studies and the reason of excluding studies.

Supplemental Figure 1. The quality of all included studies.

Supplemental Figure 2. The results of traditional meta-analysis.

Supplemental Figure 3. The comparison-adjusted funnel plot.

Supplemental Table 1. The definition of major bleeding in individual studies.

Supplemental Table 2. Assessing the inconsistency based on the node-splitting

method.

Supplemental Table 3. The results of network meta-regression.

Supplemental Appendix. The references of including studies and the reason of excluding studies.

The references of including studies[1-37] were as follow:

1. Agnelli G, Buller HR, Cohen A, et al; Oral apixaban for the treatment of acute venous thromboembolism. N Engl J Med 2013; 369 (9):799-808.
2. Agnelli G, Buller HR, Cohen A, et al; Apixaban for extended treatment of venous thromboembolism. N Engl J Med 2013; 368 (8):699-708.
3. Chung N, Jeon HK, Lien LM, et al; Safety of edoxaban, an oral factor Xa inhibitor, in Asian patients with non-valvular atrial fibrillation. Thromb Haemost 2011; 105 (3):535-544.
4. Connolly SJ, Eikelboom J, Joyner C, et al; Apixaban in patients with atrial fibrillation. N Engl J Med 2011; 364 (9):806-817.
5. Connolly SJ, Ezekowitz MD, Yusuf S, et al; Dabigatran versus warfarin in patients with atrial fibrillation. N Engl J Med 2009; 361 (12):1139-1151.
6. Eikelboom JW, Connolly SJ, Bosch J, et al; Rivaroxaban with or without Aspirin in Stable Cardiovascular Disease. N Engl J Med 2017; 377 (14):1319-1330.
7. Eriksson BI, Borris LC, Friedman RJ, et al; Rivaroxaban versus enoxaparin for thromboprophylaxis after hip arthroplasty. N Engl J Med 2008; 358 (26):2765-2775.
8. Fuji T, Fujita S, Kawai Y, et al; Efficacy and safety of edoxaban versus enoxaparin for the prevention of venous thromboembolism following total hip arthroplasty: STARS J-V. Thromb J 2015; 13:27.
9. Fuji T, Fujita S, Kawai Y, et al; Safety and efficacy of edoxaban in patients undergoing hip fracture surgery. Thromb Res 2014; 133 (6):1016-1022.
10. Fuji T, Wang CJ, Fujita S, et al; Safety and efficacy of edoxaban, an oral factor Xa inhibitor, versus enoxaparin for thromboprophylaxis after total knee arthroplasty: the STARS E-3 trial. Thromb Res 2014; 134 (6):1198-1204.
11. Giugliano RP, Ruff CT, Braunwald E, et al; Edoxaban versus warfarin in patients with atrial fibrillation. N Engl J Med 2013; 369 (22):2093-2104.
12. Granger CB, Alexander JH, McMurray JJ, et al; Apixaban versus warfarin in patients with atrial fibrillation. N Engl J Med 2011; 365 (11):981-992.
13. Hokusai VTEI, Buller HR, Decousus H, et al; Edoxaban versus warfarin for the treatment of symptomatic venous thromboembolism. N Engl J Med 2013; 369 (15):1406-1415.
14. Hori M, Matsumoto M, Tanahashi N, et al; Rivaroxaban vs. warfarin in Japanese patients with atrial fibrillation - the J-ROCKET AF study. Circ J 2012; 76 (9):2104-2111.
15. Investigators E, Bauersachs R, Berkowitz SD, et al; Oral rivaroxaban for symptomatic venous thromboembolism. N Engl J Med 2010; 363 (26):2499-2510.
16. Kakkar AK, Brenner B, Dahl OE, et al; Extended duration rivaroxaban versus short-term enoxaparin for the prevention of venous thromboembolism after total hip arthroplasty: a double-blind, randomised controlled trial. Lancet 2008; 372 (9632):31-39.

17. Lassen MR, Gallus A, Raskob GE, et al; Apixaban versus enoxaparin for thromboprophylaxis after hip replacement. N Engl J Med 2010; 363 (26):2487-2498.
18. Lassen MR, Raskob GE, Gallus A, et al; Apixaban versus enoxaparin for thromboprophylaxis after knee replacement (ADVANCE-2): a randomised double-blind trial. Lancet 2010; 375 (9717):807-815.
19. Lassen MR, Raskob GE, Gallus A, et al; Apixaban or enoxaparin for thromboprophylaxis after knee replacement. N Engl J Med 2009; 361 (6):594-604.
20. Patel MR, Mahaffey KW, Garg J, et al; Rivaroxaban versus warfarin in nonvalvular atrial fibrillation. N Engl J Med 2011; 365 (10):883-891.
21. Schulman S, Kakkar AK, Goldhaber SZ, et al; Treatment of acute venous thromboembolism with dabigatran or warfarin and pooled analysis. Circulation 2014; 129 (7):764-772.
22. Schulman S, Kearon C, Kakkar AK, et al; Dabigatran versus warfarin in the treatment of acute venous thromboembolism. N Engl J Med 2009; 361 (24):2342-2352.
23. Schulman S, Kearon C, Kakkar AK, et al; Extended use of dabigatran, warfarin, or placebo in venous thromboembolism. N Engl J Med 2013; 368 (8):709-718.
24. Turpie AG, Lassen MR, Davidson BL, et al; Rivaroxaban versus enoxaparin for thromboprophylaxis after total knee arthroplasty (RECORD4): a randomised trial. Lancet 2009; 373 (9676):1673-1680.
25. Calkins H, Willems S, Gerstenfeld EP, et al; Uninterrupted Dabigatran versus Warfarin for Ablation in Atrial Fibrillation. N Engl J Med 2017; 376 (17):1627-1636.
26. Camporese G, Bernardi E, Noventa F, et al; Efficacy of Rivaroxaban for thromboprophylaxis after Knee Arthroscopy (ERIKA). A phase II, multicentre, double-blind, placebo-controlled randomised study. Thromb Haemost 2016; 116 (2):349-355.
27. Cappato R, Ezekowitz MD, Klein AL, et al; Rivaroxaban vs. vitamin K antagonists for cardioversion in atrial fibrillation. Eur Heart J 2014; 35 (47):3346-3355.
28. Diener HC, Sacco RL, Easton JD, et al; Dabigatran for Prevention of Stroke after Embolic Stroke of Undetermined Source. N Engl J Med 2019; 380 (20):1906-1917.
29. Eriksson BI, Borris L, Dahl OE, et al; Oral, direct Factor Xa inhibition with BAY 59-7939 for the prevention of venous thromboembolism after total hip replacement. J Thromb Haemost 2006; 4 (1):121-128.
30. Ezekowitz MD, Pollack CV, Jr., Halperin JL, et al; Apixaban compared to heparin/vitamin K antagonist in patients with atrial fibrillation scheduled for cardioversion: the EMANATE trial. Eur Heart J 2018; 39 (32):2959-2971.
31. Goette A, Merino JL, Ezekowitz MD, et al; Edoxaban versus enoxaparin–warfarin in patients undergoing cardioversion of atrial fibrillation (ENSURE-AF): a randomised, open-label, phase 3b trial. The Lancet 2016; 388 (10055):1995-2003.
32. Hart RG, Sharma M, Mundl H, et al; Rivaroxaban for Stroke Prevention after Embolic Stroke of Undetermined Source. N Engl J Med 2018; 378 (23):2191-2201.
33. Mikulik R, Eckstein J, Pearce LA, et al; Frequency and Predictors of Major Bleeding in Patients With Embolic Strokes of Undetermined Source: NAVIGATE-ESUS Trial. Stroke 2020; 51 (7):2139-2147.
34. Nakamura M, Nishikawa M, Komuro I, et al; Apixaban for the Treatment of Japanese Subjects With Acute Venous Thromboembolism (AMPLIFY-J Study). Circ J 2015;

79 (6):1230-1236.
35. Ogawa S, Shinohara Y, Kanmuri K; Safety and efficacy of the oral direct factor xa inhibitor apixaban in Japanese patients with non-valvular atrial fibrillation. -The ARISTOTLE-J study. Circ J 2011; 75 (8):1852-1859.
36. Van Mieghem NM, Unverdorben M, Hengstenberg C, et al; Edoxaban versus Vitamin K Antagonist for Atrial Fibrillation after TAVR. N Engl J Med 2021; 385 (23):2150-2160.
37. Yamada N, Hirayama A, Maeda H, et al; Oral rivaroxaban for Japanese patients with symptomatic venous thromboembolism - the J-EINSTEIN DVT and PE program. Thromb J 2015; 13:2.

The reason of excluding studies was listed as follow: five that included cancer patients[1-5]; seven that included ACS patients[6-12]; four that included AF patients undergoing PCI[13-16]; two in which the background therapy included antiplatelet regimens[17, 18]; one in which the intervention was NOAC combined with an antiplatelet therapy[19]; one that included very elderly patients[20]; and eight that did not report major GI events[21-28].

References

1. Agnelli G, Becattini C, Meyer G, et al; Apixaban for the Treatment of Venous Thromboembolism Associated with Cancer. N Engl J Med 2020; 382 (17):1599-1607.
2. McBane RD, 2nd, Wysokinski WE, Le-Rademacher JG, et al; Apixaban and dalteparin in active malignancy-associated venous thromboembolism: The ADAM VTE trial. J Thromb Haemost 2020; 18 (2):411-421.
3. Planquette B, Bertoletti L, Charles-Nelson A, et al; Rivaroxaban vs Dalteparin in Cancer-Associated Thromboembolism: A Randomized Trial. Chest 2022; 161 (3):781-790.
4. Raskob GE, van Es N, Verhamme P, et al; Edoxaban for the Treatment of Cancer-Associated Venous Thromboembolism. N Engl J Med 2018; 378 (7):615-624.
5. Young AM, Marshall A, Thirlwall J, et al; Comparison of an Oral Factor Xa Inhibitor With Low Molecular Weight Heparin in Patients With Cancer With Venous Thromboembolism: Results of a Randomized Trial (SELECT-D). J Clin Oncol 2018; 36 (20):2017-2023.
6. Alexander JH, Lopes RD, James S, et al; Apixaban with antiplatelet therapy after acute coronary syndrome. N Engl J Med 2011; 365 (8):699-708.
7. Committee AS, Investigators, Alexander JH, et al; Apixaban, an oral, direct, selective factor Xa inhibitor, in combination with antiplatelet therapy after acute coronary syndrome: results of the Apixaban for Prevention of Acute Ischemic and Safety Events (APPRAISE) trial. Circulation 2009; 119 (22):2877-2885.
8. Mega JL, Braunwald E, Mohanavelu S, et al; Rivaroxaban versus placebo in patients with acute coronary syndromes (ATLAS ACS-TIMI 46): a randomised, double-blind, phase II trial. The Lancet 2009; 374 (9683):29-38.
9. Mega JL, Braunwald E, Wiviott SD, et al; Rivaroxaban in patients with a recent acute coronary syndrome. N Engl J Med 2012; 366 (1):9-19.
10. Ohman EM, Roe MT, Steg PG, et al; Clinically significant bleeding with low-dose rivaroxaban versus aspirin, in addition to P2Y12 inhibition, in acute coronary syndromes (GEMINI-ACS-1): a double-blind, multicentre, randomised trial. The Lancet 2017; 389 (10081):1799-1808.
11. Oldgren J, Budaj A, Granger CB, et al; Dabigatran vs. placebo in patients with acute coronary syndromes on dual antiplatelet therapy: a randomized, double-blind, phase II trial. Eur Heart J 2011; 32 (22):2781-2789.
12. Steg PG, Mehta SR, Jukema JW, et al; RUBY-1: a randomized, double-blind, placebo-controlled trial of the safety and tolerability of the novel oral factor Xa

inhibitor darexaban (YM150) following acute coronary syndrome. Eur Heart J 2011; 32 (20):2541-2554.
13. Cannon CP, Bhatt DL, Oldgren J, et al; Dual Antithrombotic Therapy with Dabigatran after PCI in Atrial Fibrillation. N Engl J Med 2017; 377 (16):1513-1524.
14. Gibson CM, Mehran R, Bode C, et al; Prevention of Bleeding in Patients with Atrial Fibrillation Undergoing PCI. N Engl J Med 2016; 375 (25):2423-2434.
15. Lopes RD, Heizer G, Aronson R, et al; Antithrombotic Therapy after Acute Coronary Syndrome or PCI in Atrial Fibrillation. N Engl J Med 2019; 380 (16):1509-1524.
16. Vranckx P, Valgimigli M, Eckardt L, et al; Edoxaban-based versus vitamin K antagonist-based antithrombotic regimen after successful coronary stenting in patients with atrial fibrillation (ENTRUST-AF PCI): a randomised, open-label, phase 3b trial. The Lancet 2019; 394 (10206):1335-1343.
17. Bonaca MP, Bauersachs RM, Anand SS, et al; Rivaroxaban in Peripheral Artery Disease after Revascularization. N Engl J Med 2020; 382 (21):1994-2004.
18. Zannad F, Anker SD, Byra WM, et al; Rivaroxaban in Patients with Heart Failure, Sinus Rhythm, and Coronary Disease. N Engl J Med 2018; 379 (14):1332-1342.
19. Yasuda S, Kaikita K, Akao M, et al; Antithrombotic Therapy for Atrial Fibrillation with Stable Coronary Disease. N Engl J Med 2019; 381 (12):1103-1113.
20. Okumura K, Akao M, Yoshida T, et al; Low-Dose Edoxaban in Very Elderly Patients with Atrial Fibrillation. N Engl J Med 2020; 383 (18):1735-1745.
21. Cohen AT, Spiro TE, Buller HR, et al; Rivaroxaban for thromboprophylaxis in acutely ill medical patients. N Engl J Med 2013; 368 (6):513-523.
22. Eikelboom JW, Connolly SJ, Brueckmann M, et al; Dabigatran versus warfarin in patients with mechanical heart valves. N Engl J Med 2013; 369 (13):1206-1214.
23. Ezekowitz MD, Reilly PA, Nehmiz G, et al; Dabigatran with or without concomitant aspirin compared with warfarin alone in patients with nonvalvular atrial fibrillation (PETRO Study). Am J Cardiol 2007; 100 (9):1419-1426.
24. Goldhaber SZ, Leizorovicz A, Kakkar AK, et al; Apixaban versus enoxaparin for thromboprophylaxis in medically ill patients. N Engl J Med 2011; 365 (23):2167-2177.
25. Guimaraes HP, Lopes RD, de Barros ESPGM, et al; Rivaroxaban in Patients with Atrial Fibrillation and a Bioprosthetic Mitral Valve. N Engl J Med 2020; 383 (22):2117-2126.
26. Lassen MR, Ageno W, Borris LC, et al; Rivaroxaban versus enoxaparin for thromboprophylaxis after total knee arthroplasty. N Engl J Med 2008; 358 (26):2776-2786.
27. Raskob G, Cohen AT, Eriksson BI, et al; Oral direct factor Xa inhibition with edoxaban for thromboprophylaxis after elective total hip replacement. A randomised double-blind dose-response study. Thromb Haemost 2010; 104

(3):642-649.

28. Weitz JI, Connolly SJ, Patel I, et al; Randomised, parallel-group, multicentre, multinational phase 2 study comparing edoxaban, an oral factor Xa inhibitor, with warfarin for stroke prevention in patients with atrial fibrillation. Thromb Haemost 2010; 104 (3):633-641.

Supplemental Figure 1. The quality of all included studies.

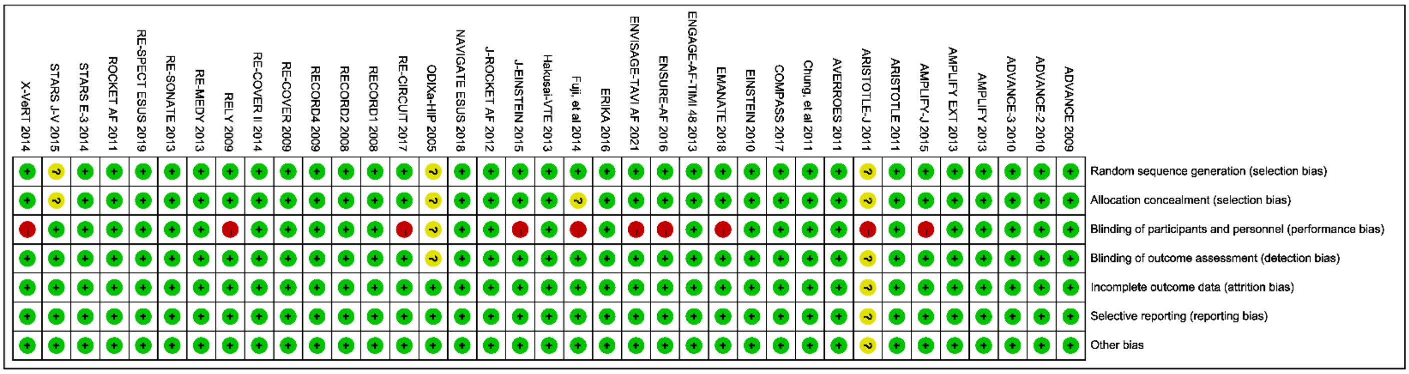

Supplemental Figure 2. The results of traditional meta-analysis.

Study	Experimental Events	Experimental Total	Control Events	Control Total	OR	95%-CI	Weight
**apixaban 5mg daily**							
ADVANCE 2009	1	1596	6	1588	0.17	[0.02; 1.37]	0.7%
ADVANCE-2 2010	1	1501	2	1508	0.50	[0.05; 5.54]	0.6%
ADVANCE-3 2010	4	2673	0	2659	8.97	[0.48; 166.62]	0.4%
AMPLIFY EXT 2013	3	840	2	829	1.48	[0.25; 8.89]	1.0%
ARISTOTLE-J 2011	0	74	0	74			0.0%
Random effects model		6684		6658	0.88	[0.20; 3.87]	2.8%
Heterogeneity: $I^2 = 44\%$, $\tau^2 = 0.9467$, $p = 0.15$							
**apixaban 10 mg daily**							
AMPLIFY EXT 2013	1	813	2	829	0.51	[0.05; 5.63]	0.6%
AMPLIFY 2013	7	2691	18	2704	0.39	[0.16; 0.93]	3.1%
ARISTOTLE 2011	105	9088	119	9052	0.88	[0.67; 1.14]	7.9%
AVERROES 2011	12	2808	14	2791	0.85	[0.39; 1.84]	3.7%
EMANATE 2018	3	735	4	721	0.73	[0.16; 3.29]	1.4%
ARISTOTLE-J 2011	0	74	0	74			0.0%
Random effects model		16209		16171	0.78	[0.57; 1.07]	16.7%
Heterogeneity: $I^2 = 0\%$, $\tau^2 = 0.0211$, $p = 0.52$							
**dabigatran etexilate 300mg daily**							
RE-COVER 2009	9	1274	5	1265	1.79	[0.60; 5.36]	2.3%
RE-COVER II 2014	6	1279	10	1289	0.60	[0.22; 1.66]	2.6%
RE-MEDY 2013	5	1430	8	1426	0.62	[0.20; 1.91]	2.2%
RE-SONATE 2013	2	681	0	662	4.87	[0.23; 101.73]	0.4%
RELY 2009	182	6076	120	6022	1.52	[1.20; 1.92]	8.2%
RE-SPECT ESUS 2019	27	2695	22	2695	1.23	[0.70; 2.16]	5.1%
RE-CIRCUIT 2017	1	317	2	318	0.50	[0.05; 5.54]	0.6%
Random effects model		13752		13677	1.27	[0.93; 1.73]	21.3%
Heterogeneity: $I^2 = 13\%$, $\tau^2 = 0.0337$, $p = 0.33$							
**dabigatran etexilate 220mg daily**							
RELY 2009	133	6015	120	6022	1.11	[0.87; 1.43]	8.0%
**edoxaban 30mg daily**							
Chung, et al 2011	0	79	1	75	0.31	[0.01; 7.79]	0.3%
ENGAGE-AF-TIMI 48 2013	129	7002	190	7012	0.67	[0.54; 0.84]	8.2%
Fuji, et al 2014	1	59	0	29	1.51	[0.06; 38.28]	0.3%
STARS E-3 2014	1	354	0	349	2.97	[0.12; 73.06]	0.3%
STARS J-V 2015	0	303	2	301	0.20	[0.01; 4.13]	0.4%
Random effects model		7797		7766	0.67	[0.54; 0.84]	9.6%
Heterogeneity: $I^2 = 0\%$, $\tau^2 = 0$, $p = 0.75$							
**edoxaban 60mg daily**							
Chung, et al 2011	0	80	1	75	0.31	[0.01; 7.69]	0.3%
ENGAGE-AF-TIMI 48 2013	232	7012	190	7012	1.23	[1.01; 1.49]	8.5%
Hakusai-VTE 2013	1	4118	2	4122	0.50	[0.05; 5.52]	0.6%
ENVISAGE-TAVI AF 2021	56	713	27	713	2.17	[1.35; 3.47]	5.9%
ENSURE-AF 2016	1	1067	1	1082	1.01	[0.06; 16.23]	0.4%
Random effects model		12990		13004	1.42	[0.88; 2.29]	15.8%
Heterogeneity: $I^2 = 36\%$, $\tau^2 = 0.1010$, $p = 0.18$							
**rivaroxaban 10mg daily**							
RECORD1 2008	2	2209	1	2224	2.01	[0.18; 22.23]	0.6%
RECORD2 2008	1	1228	0	1229	3.00	[0.12; 73.83]	0.3%
RECORD4 2009	1	1526	0	1508	2.97	[0.12; 72.88]	0.3%
COMPASS 2017	91	9117	65	9126	1.41	[1.02; 1.93]	7.4%
ODIXa-HIP 2006	0	136	1	132	0.32	[0.01; 7.95]	0.3%
ERIKA 2016	0	120	0	114			0.0%
Random effects model		14336		14333	1.41	[1.04; 1.93]	9.0%
Heterogeneity: $I^2 = 0\%$, $\tau^2 = 0$, $p = 0.86$							
**rivaroxaban≥15mg daily**							
J-ROCKET AF 2012	7	639	15	639	0.46	[0.19; 1.14]	3.0%
NAVIGATE ESUS 2018	21	3609	11	3604	1.91	[0.92; 3.97]	3.9%
EINSTEIN-Continued Treatment Study 2010	3	598	0	590	6.94	[0.36; 134.67]	0.4%
ROCKET AF 2011	224	7111	154	7125	1.47	[1.20; 1.81]	8.4%
ODIXa-HIP 2006	0	133	1	132	0.33	[0.01; 8.13]	0.3%
X-VeRT 2014	3	988	1	499	1.52	[0.16; 14.62]	0.7%
J-EINSTEIN 2015	0	47	0	12			0.0%
Random effects model		13125		12601	1.24	[0.68; 2.27]	16.7%
Heterogeneity: $I^2 = 42\%$, $\tau^2 = 0.2281$, $p = 0.13$							
**apixaban 10mg daily**							
AMPLIFY-J 2015	0	40	0	40			0.0%
**Random effects model**		**90948**		**90272**	**1.08**	**[0.89; 1.31]**	**100.0%**

Heterogeneity: $I^2 = 51\%$, $\tau^2 = 0.1015$, $p < 0.01$

Test for subgroup differences: $\chi^2_7 = 24.49$, df = 7 ($p < 0.01$)

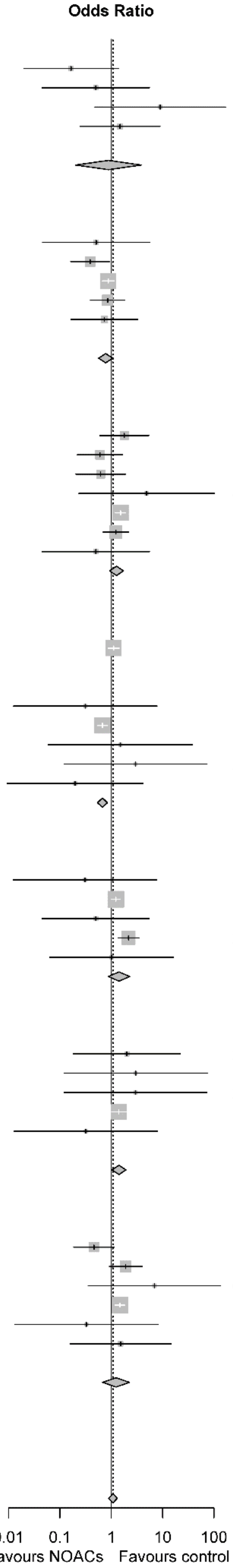

Supplemental Figure 3. The comparison-adjusted funnel plot.

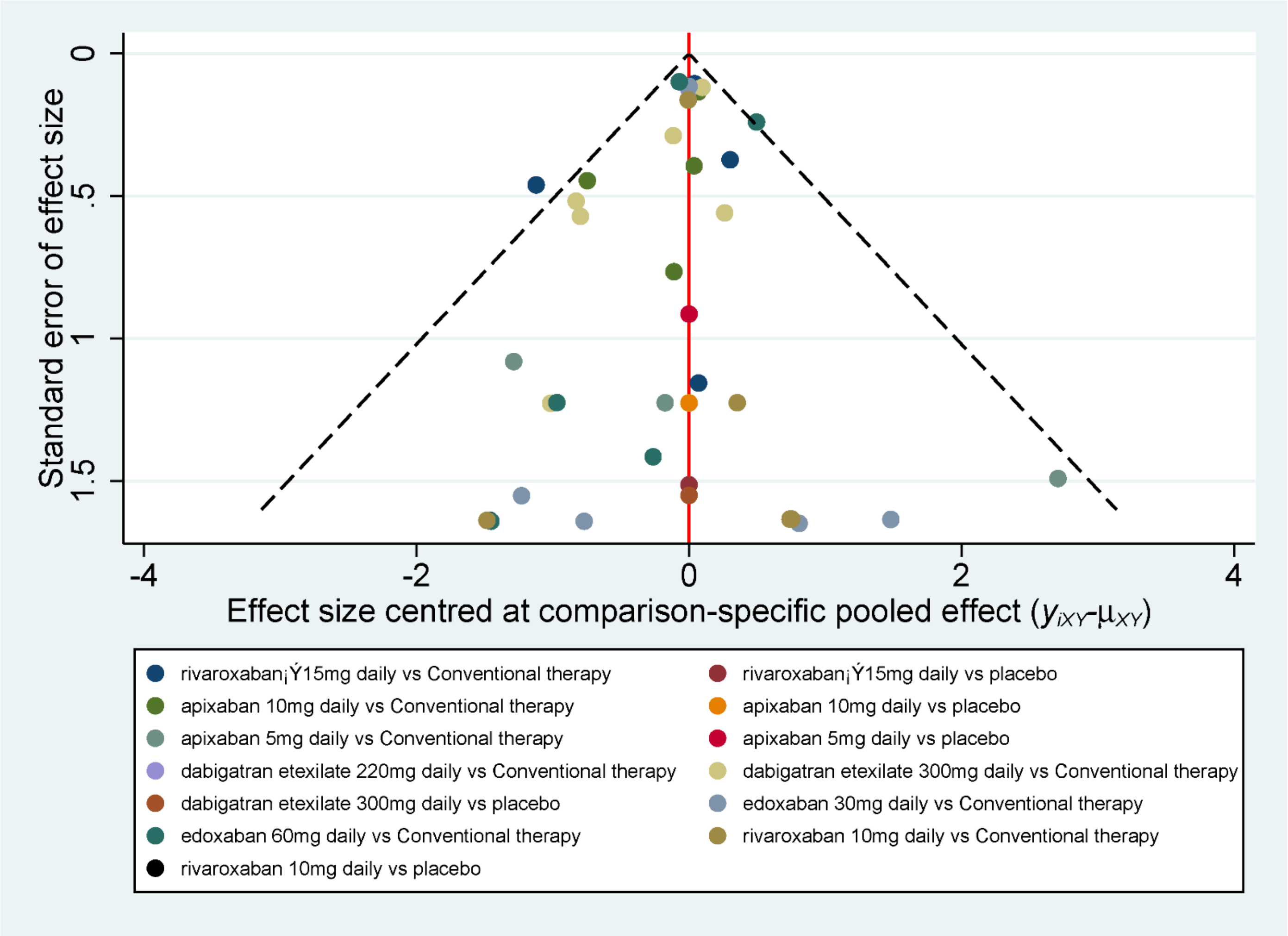

Supplemental Table 1. The definition of major bleeding in individual studies.

Study	The criteria for bleeding	Definition of major bleeding
ADVANCE 2009	ISTH	Major bleeding was defined as acute, clinically overt bleeding accompanied by one or more of the following events: a decrease in the hemoglobin level of 2 g per deciliter or more within a 24-hour period; a transfusion of 2 or more units of packed red cells; bleeding at a critical site (i.e., intracranial, intraspinal, intraocular, pericardial, or retroperitoneal bleeding); bleeding into the operated joint, requiring an additional operation or intervention; intramuscular bleeding with the compartment syndrome; or fatal bleeding.
ADVANCE-2 2010	ISTH	A decrease in blood haemoglobin concentration of 20 g/L or more during 24 h; transfusion of two or more units of packed red blood cells; critical site bleeding (including intracranial, intraspinal, intraocular, pericardial, or retroperitoneal bleeding); bleeding into the operated joint needing reoperation or intervention; intramuscular bleeding with compartment syndrome; or fatal bleeding
ADVANCE-3 2010	ISTH	The definition of major bleeding was acute, clinically overt bleeding accompanied by one or more of the following findings: a decrease in the hemoglobin level of 2 g per deciliter or more over a 24-hour period; transfusion of 2 or more units of packed red cells; bleeding at a critical site (including intracranial, intraspinal, intraocular, pericardial, and retroperitoneal bleeding); bleeding into the operated joint, necessitating reoperation or intervention; intramuscular bleeding with the compartment syndrome; or fatal bleeding.
AMPLIFY 2013	ISTH	Bleeding was defined as major if it was overt and associated with a decrease in the hemoglobin level of 2 g per deciliter or more, required the transfusion of 2 or more units of blood, occurred into a critical site, or contributed to death.
AMPLIFY EXT 2013	ISTH	Major bleeding was defined as overt bleeding that was associated with a decrease in the hemoglobin level of 2 g per deciliter or more, led to transfusion of 2 or more units of red cells, occurred in a critical site, or contributed to death.
ARISTOTLE 2011	ISTH	Clinically overt bleeding accompanied by a decrease in the hemoglobin level of at least 2 g per deciliter or transfusion of at least 2 units of packed red cells, occurring at a critical site, or resulting in death.
AVERROES 2011	ISTH	A decrease in the hemoglobin level of 2 g per deciliter or more over a 24-hour period, transfusion of 2 or more units of packed red cells, bleeding at a critical site (intracranial, intraspinal, intraocular, pericardial, intraarticular, intramuscular with compartment syndrome, or retroperitoneal), or fatal bleeding.
RE-COVER 2009	ISTH	Bleeding was defined as major if it was clinically overt and if it was associated with a fall in the hemoglobin level of at least 20 g per liter, resulted in the need for transfusion of 2 or more units of red cells, involved a critical site, or was fatal.
RE-COVER II 2014	ISTH	Bleeding was defined as major if it was clinically overt and if it was associated with a fall in the hemoglobin level of at least 20 g per liter, resulted in the need for transfusion of 2 or more units of red cells, involved a critical site, or was fatal.
RE-MEDY 2013	ISTH	Bleeding was defined as major if it was clinically overt and associated with a fall of the hemoglobin level of 20 g/L or required transfusion of at least 2 units of red cells or, involved a critical organ or was fatal.

RE-SONATE 2013	ISTH	Bleeding was defined as major if it was clinically overt and associated with a fall of the hemoglobin level of 20 g/L or required transfusion of at least 2 units of red cells or, involved a critical organ or was fatal.
RELY 2009	ISTH	Major bleeding was defined as a reduction in the hemoglobin level of at least 20 g per liter, transfusion of at least 2 units of blood, symptomatic bleeding in a critical area or organ or fatal bleeding.
Chung, et al 2011	ISTH	Major bleeding was defined as overt if it was fatal, bleeding associated with ≥2 g/dl drop in haemoglobin, transfusion ≥800 ml of packed red blood cells or whole blood, and bleeding into a critical area or organ (retroperitoneal, intracranial, intraocular, intraspinal, intra-articular or pericardial or intramuscular with compartment syndrome).
ENGAGE-AF-TIMI 48 2013	ISTH	Bleeding was defined as major if it was clinically overt and if it was associated with a fall in the hemoglobin level of at least 20 g per liter, resulted in the need for transfusion of 2 or more units of red cells, involved a critical site, or was fatal.
Fuji, et al 2014	ISTH	Major bleeding was defined as fatal bleeding, clinically overt bleeding accompanied by a decrease in hemoglobin of N2 g/dL, clinically overt bleeding requiring transfusion (excluding predonated autologous blood) with more than 4 units of blood (1 unit = approximately 200 mL), retroperitoneal bleeding, intracranial bleeding, intraocular bleeding or intrathecal bleeding, and bleeding requiring repeat surgery.
Hakusai-VTE 2013	ISTH	Bleeding was defined as major if it was overt and was associated with a decrease in hemoglobin of 2 g per deciliter or more or required a transfusion of 2 or more units of blood, occurred in a critical site, or contributed to death.
STARS E-3 2014	ISTH	Major bleeding was defined as fatal bleeding, clinically apparent bleeding with a decrease in haemoglobin of more than 2 g/dL, clinically apparent bleeding that requires transfusion of more than 4 units (1 unit = approximately 200 mL) of blood (excluding transfusion of stored autologous blood), retroperitoneal bleeding, intracranial bleeding, intraocular bleeding, or intrathaecal bleeding, or bleeding necessitating additional surgery.
STARS J-V 2015	ISTH	Major bleeding was defined as fatal bleeding; clinically overt bleeding accompanied by a decrease in hemoglobin of >2 g/dL; clinically overt bleeding requiring hemotransfusion with more than four units of blood; retroperitoneal, intracranial, intraocular, or intrathecal bleeding; or bleeding requiring repeat surgery.
COMPASS 2017	ISTH	Major bleeding was defined as fatal bleeding; clinically overt bleeding accompanied by a decrease in hemoglobin of >2 g/dL; clinically overt bleeding requiring hemotransfusion with more than four units of blood; retroperitoneal, intracranial, intraocular, or intrathecal bleeding; or bleeding requiring repeat surgery. All bleeding that led to presentation to an acute care facility or hospitalization also as major bleeding.
EINSTEIN 2010	ISTH	Bleeding was defined as major if it was clinically overt and associated with a fall in the hemoglobin level of 20 g per liter or more, or if it led to transfusion of two or more units of red cells, or if it was retroperitoneal, intracranial, occurred in a critical site, or contributed to death.
J-ROCKET AF 2012	ISTH	Clinically overt bleeding that was associated with a fall in hemoglobin ≥20 g/L, transfusion of ≥2units of packed red blood cells or whole blood, or involved a critical site

		(intracranial, intraspinal, intraocular, pericardial, intra-articular, intramuscular with compartment syndrome, retroperitoneal hemorrhage), or had a fatal outcome.
RECORD1 2008	ISTH	Major bleeding was defined as bleeding that was fatal, occurred in a critical organ (e.g., retroperitoneal, intracranial, intraocular, and intraspinal bleeding), or required reoperation or extra surgical-site bleeding that was clinically overt and was associated with a fall in the hemoglobin level of at least 2 g per deciliter or that required transfusion of 2 or more units of whole blood or packed cells.
RECORD2 2008	ISTH	Major bleeding was defined as bleeding that was fatal, was into a critical organ (eg, retroperitoneal, intracranial, intraocular, intraspinal), required re-operation, or clinically overt extra-surgical-site bleeding associated with a fall in haemoglobin of 20 g/L or more, calculated from the day 1 post-operative baseline value, or requiring infusion of two or more units of whole blood or packed cells.
RECORD4 2009	ISTH	Major bleeding was defined as clinically overt bleeding that was fatal, occurred in a critical organ (eg, retroperitoneal, intracranial, intraocular, or intraspinal), necessitated operation, was outside of the surgical site and associated with a fall in haemoglobin of 2 g/dL or more (calculated from the postoperative haemoglobin baseline value before the event), or required an infusion of two or more units of blood.
ROCKET AF 2011	ISTH	Major bleeding was defined as clinically overt bleeding associated with any of the following: fatal outcome, involvement of a critical anatomic site (intracranial, spinal, ocular, pericardial, articular, retroperitoneal, or intramuscular with compartment syndrome), fall in hemoglobin concentration >2 g/dL, transfusion of >2 units of whole blood or packed red blood cells, or permanent disability.
AMPLIFY-J 2015	ISTH	Bleeding was defined as major if it was overt and associated with a decrease in the hemoglobin level of 2 g per deciliter or more, required the transfusion of 2 or more units of blood, occurred into a critical site, or contributed to death.
ARISTOTLE-J 2015	ISTH	Major bleeds were defined as acute, clinically overt, and with 1 or more of the following: a decrease in hemoglobin ≥2 g/dl over a 24-h period; bleeding requiring transfusion of ≥2 units of packed red blood cells; or bleeding in a critical site (intracranial, intraspinal, intraocular [not conjunctival], pericardial, intra-articular, intramuscular with compartment syndrome, retroperitoneal); or bleeding that was fatal.
EMANATE 2018	ISTH	The definition of major bleeding was adapted from the International Society on Thrombosis and Haemostasis as clinically overt bleeding accompanied by one or more of the following: a decrease in haemoglobin of >_1.24mmol/L; transfusion of >_2 units of packed red blood cells; bleeding that occurs in at least one of the following critical sites: intracranial, intraspinal, intraocular, pericardial, intra-articular, intramuscular with compartment syndrome, retroperitoneal; or bleeding that is fatal.
ENSURE-AF 2016	ISTH	Bleeding was defined as major if it was clinically overt and associated with a decrease in hemoglobin levels of 2.0 g per deciliter or more or a transfusion of 2 or more units of red blood cells; or if bleeding was intracranial or retroperitoneal in nature, occurred in another critical site, or contributed to death.

ENVISAGE-TAVI AF 2021	ISTH	Bleeding was defined as major if it was clinically overt and associated with a decrease in hemoglobin levels of 2.0 g per deciliter or more or a transfusion of 2 or more units of red blood cells; or if bleeding was intracranial or retroperitoneal in nature, occurred in another critical site, or contributed to death.
ERIKA 2016	ISTH	Bleeding was defined as major if it was clinically overt and associated with a fall in the hemoglobin level of 20 g per liter or more, or if it led to transfusion of two or more units of red cells, or if it was retroperitoneal, intracranial, occurred in a critical site, or contributed to death.
J-EINSTEIN 2015	ISTH	Bleeding was defined as major if it was clinically overt and associated with a decrease in hemoglobin levels of 2.0 g per deciliter or more or a transfusion of 2 or more units of red blood cells; or if bleeding was intracranial or retroperitoneal in nature, occurred in another critical site, or contributed to death.
NAVIGATE ESUS 2018	ISTH	Bleeding was defined as major if it was clinically overt and associated with a decrease in hemoglobin levels of 2.0 g per deciliter or more or a transfusion of 2 or more units of red blood cells; or if bleeding was intracranial or retroperitoneal in nature, occurred in another critical site, or contributed to death.
ODIXa-HIP 2006	ISTH	Major bleeding was defined as bleeding starting ‡6 h after surgery, or after the first postoperative dose of study medication (whichever came first), but not >2 days after the last administration of study drug – which included: fatal bleeding; bleeding into a critical organ (retroperitoneal, intracranial, intraocular, or intraspinal bleeding); overt bleeding warranting treatment cessation; bleeding leading to re-operation; or clinically overt bleeding associated with a fall in hemoglobin of ‡2 g dL)1 or leading to transfusion of at least two units of blood.
RE-CIRCUIT 2017	ISTH	Bleeding was defined as major if it was clinically overt and associated with a decrease in hemoglobin levels of 2.0 g per deciliter or more or a transfusion of 2 or more units of red blood cells; or if bleeding was intracranial or retroperitoneal in nature, occurred in another critical site, or contributed to death.
RE-SPECT ESUS 2019	ISTH	Bleeding was defined as major if it was clinically overt and associated with a decrease in hemoglobin levels of 2.0 g per deciliter or more or a transfusion of 2 or more units of red blood cells; or if bleeding was intracranial or retroperitoneal in nature, occurred in another critical site, or contributed to death.
X-VeRT 2014	ISTH	Bleeding was defined as major if it was clinically overt and associated with a decrease in hemoglobin levels of 2.0 g per deciliter or more or a transfusion of 2 or more units of red blood cells; or if bleeding was intracranial or retroperitoneal in nature, occurred in another critical site, or contributed to death.

Supplemental Table 2. Assessing the inconsistency based on the node-splitting method.

Comparison	P value
apixaban 5mg daily vs. placebo	0.93
apixaban 5mg daily vs. apixaban 10mg daily	0.50
apixaban 5mg daily vs. conventional therapy	0.59
apixaban 10mg daily vs. placebo	0.35
dabigatran etexilate 300mg daily vs. placebo	0.64
rivaroxaban 10mg daily vs. placebo	0.53
rivaroxaban≥15mg daily vs. placebo	0.43
apixaban 10mg daily vs. conventional therapy	0.29
dabigatran etexilate 300mg daily vs. conventional therapy	0.70
edoxaban 30mg daily vs. edoxaban 60mg daily	0.77
rivaroxaban 10mg daily vs. rivaroxaban≥15mg daily	0.97
rivaroxaban 10mg daily vs. conventional therapy	0.49
rivaroxaban≥15mg daily vs. conventional therapy	0.43

Supplemental Table 3. The results of network meta-regression.

Variables	interaction term
Age	0.619(-0.076—1.335)
Male	-0.310(-0.923—0.291)
BMI	-0.624(-1.612—0.343)
Weight	-0.597(-2.087—0.719)
Asian	-0.519(-1.111—0.045)
Indication	0.322(-0.065—0.697)
Follow-up time	0.005(-0.336—0.348)
